# Variability of Properties Modulating the Biosynthesis of Biologically Active Compounds in Young Barley Treated with Ozonated Water

**DOI:** 10.3390/molecules28135038

**Published:** 2023-06-27

**Authors:** Natalia Matłok, Tomasz Piechowiak, Ireneusz Kapusta, Radosław Józefczyk, Maciej Balawejder

**Affiliations:** 1 Department of Food and Agriculture Production Engineering, University of Rzeszow, St. Zelwerowicza 4, 35-601 Rzeszow, Poland; 2 Department of Chemistry and Food Toxicology, University of Rzeszow, St. Ćwiklińskiej 1a, 35-601 Rzeszow, Poland; tpiechowiak@ur.edu.pl (T.P.); rjozefczyk@ur.edu.pl (R.J.); mbalawejder@ur.edu.pl (M.B.); 3 Department of Food Technology and Human Nutrition, Rzeszow University, St. Zelwerowicza 4, 35-601 Rzeszow, Poland; ikapusta@ur.edu.pl

**Keywords:** ozone, water, young barley leaves, polyphenols, UPLC–PDA–MS/MS, antioxidant activities, ascorbic acid content, reactive oxygen species generation, superoxide dismutase, catalase activity, phenylalanine ammonia lyase activity, polyphenol oxidase activity

## Abstract

This paper presents the effects of irrigating barley plants with different type of water solutions saturated with gaseous ozone generated from atmospheric air. The study investigated the effects of the applied types of water on the modulation of the biosynthesis of selected bioactive compounds (content of total polyphenols, small molecule antioxidants, vitamin C) in the produced plant material. A number of transformations of reactive oxygen species (ROS) and nitrogen compounds have also been postulated; these are observed during the saturation of water with gaseous O_3_ and 30 min after the end of the process. It was shown that after the process of water saturation with gaseous O_3_, the gas later is converted to compounds with high oxidative potential and good stability; these, in turn, lead to the oxidation of oxidates generated from atmospheric nitrogen into nitrates, which exhibit fertilising properties. Thirty minutes after the process of H_2_O saturation with gaseous O_3_ was completed, the tests showed the highest concentrations of nitrates and the relatively high oxidative potential of the solution originating from H_2_O_2_ with a low concentration of the dissolved O_3_. This solution exhibited the highest activity modulating the biosynthesis of polyphenols, small molecule antioxidants and vitamin C in young barley plants. The resulting differences were significant, and they were reflected by 15% higher total polyphenol content, 35% higher antioxidative potential and 57% greater content of vitamin C compared to the control specimens (plants treated with fresh H_2_O).

## 1. Introduction

Different plant materials are valuable sources of nutrients and bioactive compounds. Valuable plant materials with significant bioactive substance contents include seedlings of young plants, such as barley (*Hordeum vulgare* L.) [1,2]. The latter material is used in functional foods due to its high content of polyphenols, mainly including the following phenolic compounds: 3-*O*-feruloylquinic acid, isoorientin-7-*O*-glucoside (lutonarin), isovitexin-7-*O*-glucoside (saponarin), isovitexin, isoorientin-7-*O*-[6-feruloyl]-*β*-glucopyranoside and isooreintin-7-*O*-[6-sinapoyl]-*β*-glucopyranoside [3,4]. Moreover, young barley plants contain significant amounts of vitamins B, C, E and K; iron; magnesium; calcium; amino acids and fibre [4,5].

The contents of bioactive compounds in plant materials can be modified in growing plants by applying various abiotic factors. These include fertilisation, fumigation, intensity of light and temperature, as well as moisture and type of soil [6]. The level of plant nutrition affects the intensity of photosynthesis and total metabolism, which directly translates into the level of ROS and induces the level of activity of the oxidant scavenging system [7,8,9,10]. In plant cultivation, water is a carrier of nutrients [11]. Nutrients can be provided to plants through soil fertilisation and in a form of a solution. This is accomplished by using foliar sprays, fertigation (hydroponic cultivation) and watering [11,12,13].

The properties of the liquid used for watering can be modified by applying ozone treatment [14]. This process was used to produce tomato seedlings. The use of ozonated water has been demonstrated to impact the activity of nutrients applied to fertilise plants during their growth. The ozone source used generated a mixture of ozone and molecular oxygen (O_2_), which resulted from the fact that input gas material was provided to the generator from an oxygen concentrator. The use of water saturated with ozone had a positive effect on the biometric parameters of tomato plants [15]. Ozone and ozonated water can have a positive impact on the microbiological status of young plants. It is a factor with one of the smallest molecular masses and high activity, while other factors, including furochromone derivatives, are equally active, but their total synthesis is complicated [16,17,18]. 

Systems supplied with atmospheric air are more commonly used to obtain ozone. These generate lower concentrations of gaseous O_3_, with relatively high flow rates. As was shown in [19], when ozone is generated from air, nitrogen oxides with varied oxidation states are also produced. This mixture leads to similar effects as those observed in the case of photochemical smog. These are manifested by necroses and other changes typically occurring in plants exposed to ozone or acid rain [20]. Historically, oxidation of atmospheric nitrogen was applied in the production of nitric acid, which is one of the main components used in fertiliser manufacturing [21]. Nitrogen in the form of nitrate is an easily available building component used by plants, and its supply also translates into the content of bioactive compounds in tissues.

This paper presents the effects of treating young barley plants with different types of water solutions saturated with gaseous ozone generated from atmospheric air. The study investigated the effects of the applied types of water on the modulation of the biosynthesis of selected bioactive compounds (content of total polyphenols, small molecule antioxidants, vitamin C) in the produced plant material. It was demonstrated that these changes are mainly produced by a number of transformations of reactive oxygen species (ROS) and nitrogen compounds observed during the saturation of water with gaseous O_3_ and 30 min after the end of the process.

## 2. Results and Discussion

Water is a carrier of minerals necessary for plant growth and development. However, during the irrigation process, a number of pathogens are transmitted, mainly bacteria and fungi causing various diseases [22,23,24]. The problem may be reduced or eliminated by applying ozone treatment to plants [7,9] or to H_2_O used in irrigation of plants or for preparing working fluids for crop protection treatments [25,26,27]. Ozone, as an agent with high oxidative potential, reduces the occurrence of pathogens. It is commonly used because of its short half-life and activity that does not produce residue [28]. It is assumed to decompose to atmospheric O_2_ after a few hours. In-depth analysis of the process in which ozone was applied to saturate H_2_O suggests that conversion of ozone to other reactive forms with greater stability is possible.

The experimental process of young barley production applied irrigation with three types of H_2_O, i.e., fresh water (V1), water saturated with O_3_ to a level of 3.0 ppm (V2) and water left for 30 min after the completed process of saturation with O_3,_ still containing 0.5 ppm of dissolved ozone (V3) (Table 1).

**Figure 1:**
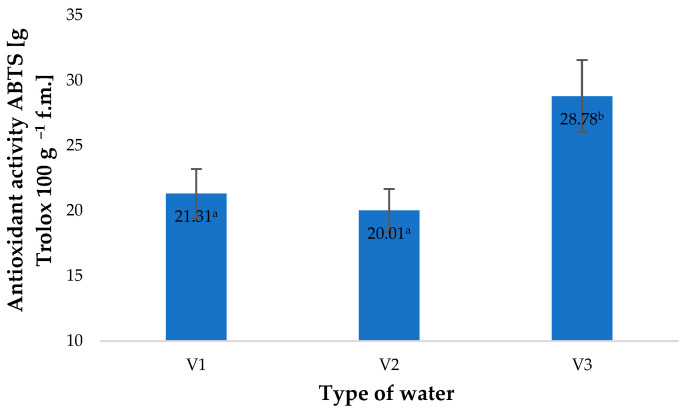
Antioxidant activity of ABTS (2.2′-azino-bis-(3-ethylbenzothiazolin-6-sulfonic acid) test in young barley plants depending on the type of water used for watering plants (*n* = 20); differences between the results are marked with small letters; significance level is defined as *p* < 0.05.

The raw material acquired after harvest was assessed for the content of selected bioactive compounds, mainly small molecule antioxidants. The highest values of antioxidant potential were found in young barley leaves acquired from raw plants watered with V3. The antioxidant potential of these plants was 35% higher compared to the control specimens (plants watered with V1) (Figure 1). Similar observations were reported when ozone solutions or gaseous ozone were applied in plant production [4,9,29]. Enhanced antioxidant activity affects the potential of young barley leaves and products derived from them. Gromkowska-Kępa et al. demonstrated that barley leaves rich in antioxidants exhibit high protective activity against high-energy UV radiation acting on human skin fibroblasts [30]. Ali et al. (2014) conditioned fresh papaya fruit by applying fumigation with gaseous O_3_ (0, 1.5, 2.5, 3.5 and 5 ppm) continuously for 96 h before the material was stored at 25 °C. After 10 days of storage, they found that the fruit exposed to O_3_ at a rate of 2.5 ppm presented 30.9% higher antioxidant activity compared to the material which was not treated with ozone [31]. Increased antioxidant potential was also observed in *Origanum majorana* L. plants by Matłok et al. [9]. This effect, however, depended on the conditions of the fumigation applied, mainly the concentration of O_3_ and duration of plant exposure to its activity. Surprisingly, the highest increase was observed in the case of water with an O_3_ concentration of 0.5 ppm (spectrophotometric). This suggests that, besides ozone itself, the antioxidant potential of the plant material produced was affected by other factors resulting from the saturation of water with O_3_.

**Figure 2:**
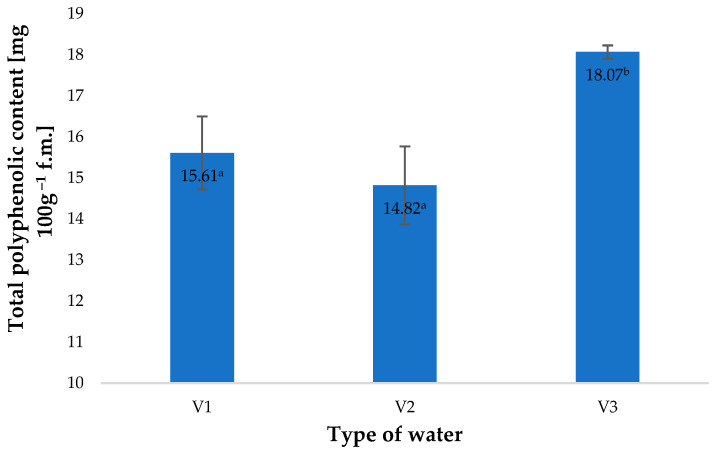
Total polyphenolic content in young barley plants depending on the type of water used for watering plants (*n* = 20); differences between the results are marked with small letters; significance level is defined as *p* < 0.05.

A similar effect of the types of water applied was observed in the total polyphenol contents of young barley leaves (Figure 2). As shown by Matłok et al. [9], if various factors stimulating the biosynthesis of polyphenols are applied, the contents of these compounds correlate with the antioxidant potential of the plant material and with the conditions used. The total content of polyphenols in control specimens (V1) amounted to 15.61 mg 100 g^−1^ of the raw material and was 15% lower than the total polyphenol content in plants watered with V3. This response of the plants was associated with an impact of similar factors as those identified in the case of antioxidant potential (Figure 1).

Total polyphenol content in raw plant material corresponds to the proportional concentrations of the specific phenolic compounds. In some cases, the percent rates of these compounds in the mixture do not change in response to the factors affecting total polyphenol content. Matłok et al. [4] reported that fumigation of barley plants with gaseous ozone did not affect the qualitative composition of polyphenols in the material. Brauch et al. demonstrated that polyphenolic profiles vary depending on the stage of barley plant growth; however, irrespective of the growth stage, the main polyphenol contained in this plant material is isovitexin 7-*O*-glucoside [32]. Similar results were observed when ozone-treated water was used [Table 2]. The mixture of polyphenols was also found with the highest proportional content of isovitexin 7-*O*-glucoside, on average amounting to 85.8%. Owing to the high content of this substance, this raw material can potentially be used as a natural component of hepatoprotective agents [33]. 

**Figure 3:**
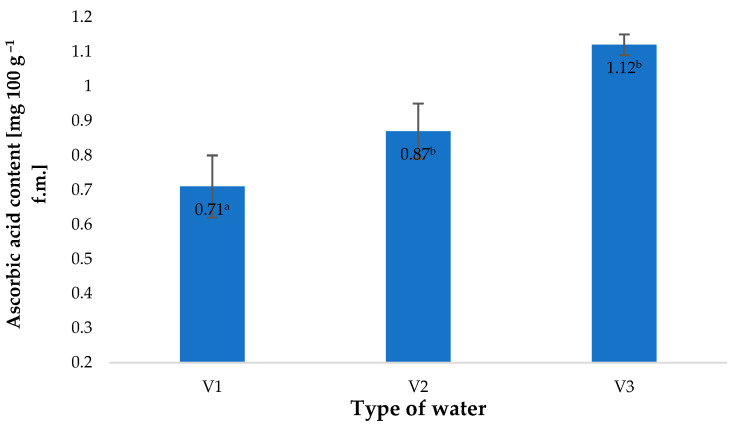
Ascorbic acid content in young barley plants depending on the type of water used for watering plants (*n* = 20); differences between the results are marked with small letters; significance level is defined as *p* < 0.05.

The types of water used in the irrigation of growing barley plants affected the contents of vitamin C in the material investigated (Figure 3). The latter value was closely correlated to the amount of nitrate anions in the types of water used. The highest vitamin C content, found in the plants watered with V3, was 57% higher than in the control sample (V1). A similar correlation was observed in the contents of nitrate anions in water. This value in V3 was 224% higher than in fresh water (V1). The relationship between the vitamin C content of the plant material produced and the amount of nitrogen supplied to the plants has also been confirmed by other researchers. Tuncay et al. [34] showed that the content of this component closely depended on the dose of nitrogen fertiliser applied.

Changes in the content of bioactive compounds in plant material are induced by the activation of the enzyme systems responsible for their biosynthesis in plants [35,36]. This activity can be stimulated during plant growth and development by applying fertilisers or other biotic and abiotic factors. These include substances with oxidising properties applied during plant growth, such as hydrogen peroxide and gaseous ozone and its solutions [37,38].

**Figure 4:**
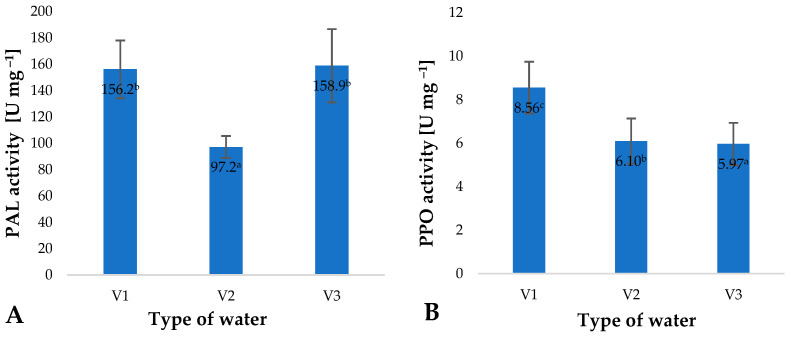
Phenylalanine ammonia lyase (PAL) (**A**) and polyphenol oxidase (PPO) (**B**) activity in young barley plants depending on the type of water used for watering plants (*n* = 20); differences between the results are marked with small letters; significance level is defined as *p* < 0.05.

The increase in the total polyphenol contents of young barley leaves, identified by the assays, resulted from the activation of phenylalanine ammonia lyase (PAL) (Figure 4A). This enzyme is involved in the biosynthesis of polyphenol precursors, mainly cinnamic acid [39]. A high level of polyphenols maintained in the plant material promotes a reduction in the activity of polyphenol oxidase (PPO) (Figure 4B). This enzyme promotes the oxidative polymerisation of polyphenols, mainly observed in enzymatic browning. Owing to the low activity of this enzyme, the raw material can effectively be used in the food and pharmaceutical industries.

The largest changes in the activity of PAL and PPO were recorded in the leaves of young barley watered with V3. This is an interesting observation given the content of ozone dissolved in the water applied. Similar effects of changes in PAL and PPO enzyme activity on the polyphenol content of the raw material produced have also been reported by other researchers. Migut et al. found that the use of antioxidants in different forms affects the activity of various enzyme systems, including PPO, which is directly reflected in reduced polyphenol degradation in maize plants [40].

In many cases, the activity of PAL and PPO enzymes is linked to the effectiveness of the enzymatic free-radical scavenging apparatus. The activity of this apparatus results from the exposure of growing plants to various factors or from increased metabolic activity. Such factors include ozone or its water solutions applied in the cultivation of plants [41]. Ozone, to a limited extent, can penetrate plant cells through the stomata or root system and subsequently can be converted to reactive oxygen species (ROS).

**Figure 5:**
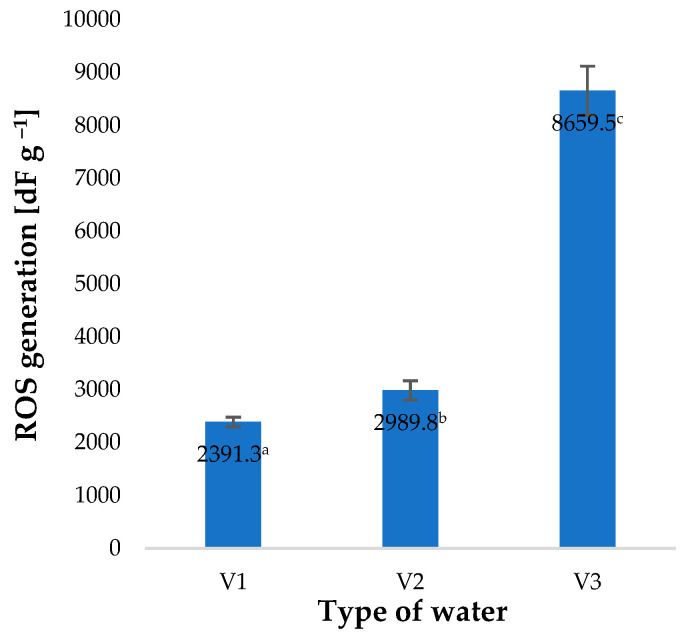
ROS generation in young barley plants depending on the type of water used for watering plants (*n* = 20); differences between the results are marked with small letters; significance level is defined as *p* < 0.05.

Irrigation of barley plants with water used immediately after the process of saturation with O_3_ (V2) was completed, producing only a small increase in ROS generated (Figure 5). Surprisingly, it was found that irrigation with water used 30 min after the completed ozone treatment (V3), with an O_3_ content of 0.5 ppm (determined by a spectrophotometric method), produced a 262% increase in the quantity of ROS generated compared to the control sample (plants watered with V1). These findings suggest that, in addition to O_3_, the solution contains other substances affecting the intensity of ROS generation [42]. The effect of various abiotic factors used during plant cultivation on the level of ROS generated has also been reported by other researchers. Balawejder et al. found that the use of different types of substrates and fertigation in the protected cultivation of raspberries affects the level of ROS generation in the plants, as well as the efficiency of the free-radical scavenging apparatus [12]. On the other hand, Huang et al. showed that various abiotic factors affect the regulation of ROS levels and their degradation mechanisms [43].

**Figure 6:**
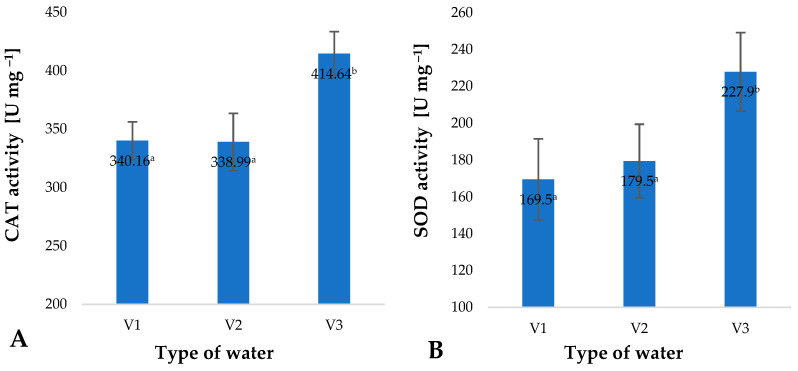
Catalase (CAT) (**A**) and superoxide dismutase (SOD) (**B**) activity in young barley plants depending on the type of water used for watering plants (*n* = 20); differences between the results are marked with small letters; significance level is defined as *p* < 0.05.

Increased intensity of ROS generation is linked with a change in the activity of enzymes responsible for free-radical scavenging. These enzymes include catalase (CAT) and superoxide dismutase (SOD). The highest activity of these enzymes was identified in the case of plant material which during the production process was irrigated with V3 (Figure 6). Activity of catalase (CAT) in plants watered with V3 was 21% higher than in the control specimens (V1) (Figure 6A). A similar change was identified in the case of superoxide dismutase (SOD), which was found with an increase of 34% (Figure 6B). Analysis of all the factors investigated, i.e., content of small molecule antioxidants, polyphenols, vitamin C and the activity of selected enzymes, shows that the plants are in a state of controlled oxidative stress. The fact that oxidative stress is observed supports the conclusion that the water used in irrigation contains other substances and factors which induce this type of stress. A similar response to stressful factors was observed in the case of the action of gaseous ozone on barley plants [4]. The highest dose of stressful factors resulted in the highest level of ROS generation. Matlok et al. suggest that this response is induced by ozone as well as other factors, including H_2_O_2_ [4].

**Figure 7:**
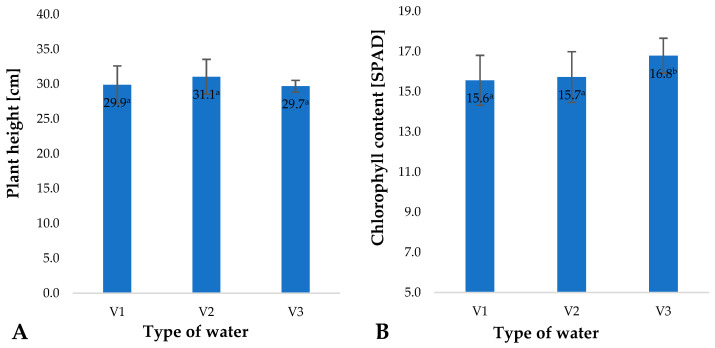
Plant height (**A**) and chlorophyll content (**B**) in young barley leaves depending on the type of water used for watering plants (*n* = 20); differences between the results are marked with small letters; significance level is defined as *p* < 0.05.

**Figure 8:**
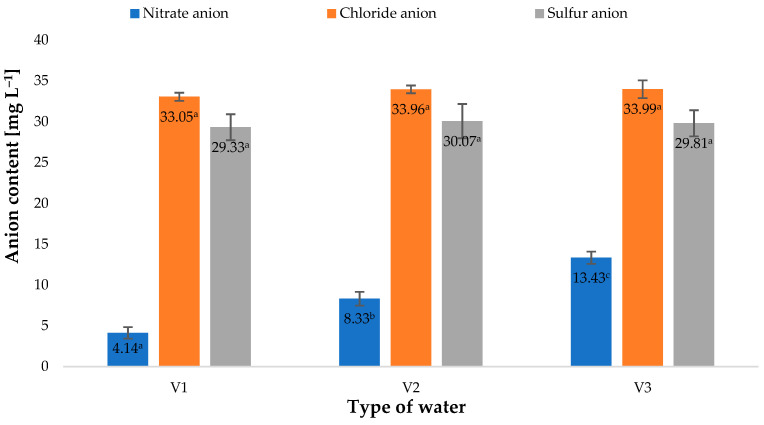
Nitrate anion. chloride anion and sulfur anion content in water (*n* = 3); differences between the results are marked with small letters; significance level is defined as *p* < 0.05.

Factors applied during plant cultivation, mainly including fertilisation and fertigation, impact not only the size of the crop, but also selected physiological parameters of plants during their growth and development [44,45]. Relative chlorophyll content in plant leaves is a basic physiological parameter reflecting the condition and nutritional status of plants. This value is mainly correlated to the applied nitrogen fertilization [10]. The types of water used in the irrigation of young barley impacted relative chlorophyll contents in the plant leaves (Figure 7B). The highest value of this parameter was found in plants irrigated with V3. This increase may be linked to the significantly higher concentration of nitrate anions in this type of water (Figure 8); owing to their fertilising properties, they beneficially affected the nutritional status of barley plants, as reflected by the SPAD measured.

At the same time, it should be noted that the water types used did not significantly affect the height of the barley plants (Figure 7A), which, with equal plant density, corresponds to the quantity of biomass produced. It should be noted that barley plants were harvested at an early stage of development (plant height of 10 cm), when—in terms of biomass growth—they mainly rely on substances stored in the caryopses. Kania et al. found that the yield of young barley production is closely dependent on the harvest date. It has also been demonstrated that the harvest date affects the quality of the raw material and the composition of the obtained juice. Changes primarily occur in protein content, pH and pigment content, mainly chlorophyll [46]. At this stage, they mainly take up water from the substrate, and in this case, the nutrients supplied with water only affected the quality of the raw material produced, not its yield. Indeed, it has been shown by many researchers that in the early stages of growth and development following seed germination, the substances stored in the caryopsis provide nourishment for the plant [47].

The response of young barley plants suggests there were considerable differences in the composition of water used for irrigation (Figure 8). An interesting fact was the difference observed in the biochemical parameters of plants irrigated with water saturated with O_3_, used immediately after the process or 30 min later. As shown in Figure 8, there was a change in the concentration of nitrate anions. Notably, forms of nitrogen could be supplied to the solution only during the saturation of H_2_O with gaseous O_3_ (Equations (1)–(4)) [48,49]:

N + O_3_ → NO + O_2_ (1)

NO + O_3_ → O_2_ + NO_2_ (2)

NO_2_ + O_3_ → O_2_ + NO_3_ (3)

NO_3_ + NO_2_ + M→N_2_O_5_ + M (4)

**Figure 9:**
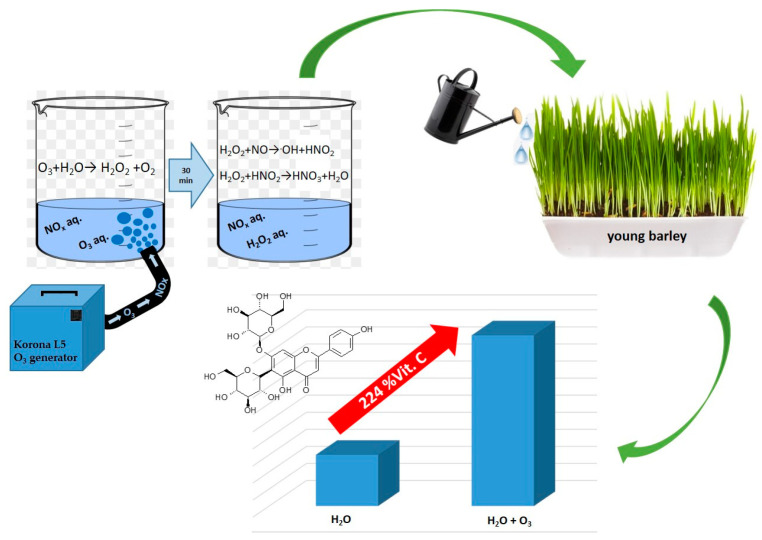
Scheme of the mechanism for modulating the biosynthesis of bioactive compounds in young barley irrigated with ozonated water.

**Figure 10:**
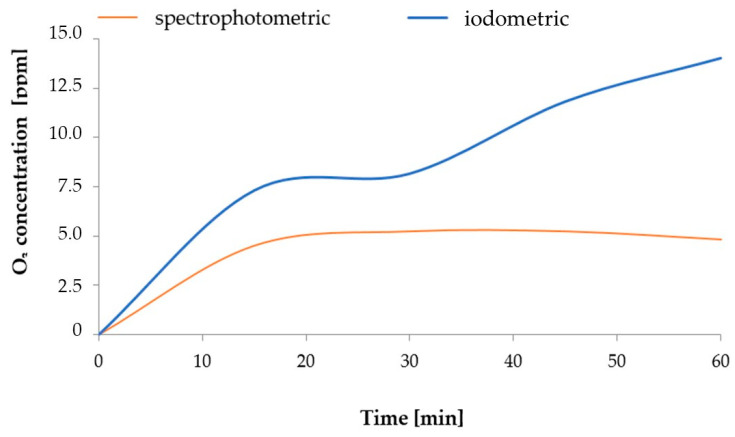
The process of water saturation with ozone.

Presumably, during this process, the stream of ozone generated from the air contained nitrogen oxides, with different oxidation states, that dissolved in H_2_O. The process of generating nitrogen oxides at the stage of ozone production from air, described in a study [50], took a course corresponding to the equations discussed by Dreschoff et al. [51]. The nitrogen oxides generated in the solution were subsequently oxidised. This process continued after the process of saturating H_2_O with gaseous O_3_ was completed. As shown in Figure 9, the quantity of dissolved ozone does not correspond to the oxidation potential, which was determined using the iodometric method. This is associated with the conversion of O_3_ dissolved in H_2_O into various reactive oxygen species. As shown by Dreschoff [51], some of these ROS are short-lived. However, hydrogen peroxide, generated in large quantity in the processes of ozone conversion and recombination of hydroxyl radicals, is responsible for long-lasting oxidative potential (Figure 10). Hydrogen peroxide, an agent with high oxidative potential, converts nitrogen oxides to the highest oxidation state (Figure 9) (Equations (5)–(8) [19,52,53,54,55]). These forms of nitrogen are most readily absorbed by plants. This is reflected by the identified relative chlorophyll content of young barley leaves, which is an indicator of the plant’s nutritional status regarding nitrogen (Figure 8B). The quantity of nitrogen absorbed by plants is related to the content of selected biologically active components, as established by Strzemski et al. [52]. However, this effect is enhanced by the presence of vaporiser. This is suggested by the identified ROS content.

O_3_ + H_2_O → H_2_O_2_ + O_2_ (5)

^•^OH + ^•^OH → H_2_O_2_ (6)

H_2_O_2_ + NO → ^•^OH + HNO_2_ (7)

H_2_O_2_ + HNO_2_ → HNO_3_ + H_2_O (8)

## 3. Materials and Methods

### 3.1. Pot Experiment

A pot experiment involving the cultivation of young barley plants (*Hordeum vulgare* L.) was conducted in controlled conditions (air temperature 20 °C, humidity 80%). Barley seeds were planted in soil which was described in detail by Matłok et al. [56]. Irrigation of the growing plants was carried out using fresh water (V1), water saturated with gaseous O_3_ immediately after the process was completed (V2) or water saturated with gaseous O_3_ 30 min after the process was completed (V3). Plant watering was performed every 24 h, and the process was designed to increase moisture level to 60% water holding capacity (WHC). The experiment was conducted in three replications, with 12 pots applied for each variant. The experiment was continued until the plants were 11.3 ± 1.8 cm in height. Subsequently, the overground biomass was cut down and subjected to analyses in order to identify the effects of the watering on the biosynthesis of selected biologically active compounds in the raw material.

### 3.2. Saturation of Water with Ozone

The process of H_2_O saturation with gaseous O_3_ was carried out using a 2 dm^3^ absorption column. The absorber was made of ozone-resistant material (PVC). Dozens of rings, each with a diameter of 1.5 cm, were placed inside the counter reactor. The rings were also made from ozone resistant material—EPDM (rubber). They allowed for better transfer of mass between water and the gaseous phase. Ozone was obtained using a Korona L5 *Zdrowa żywność* generator with a capacity of 3 g O_3_ h^−1^ and input atmospheric air at a flow rate of 10 dm^3^ min^−1^. Excess ozone, which was not absorbed, was led to the extraction installation.

### 3.3. Measurement of O_3_ Concentration in H_2_O

#### 3.3.1. Iodometric Method

In order to determine the changes in O_3_ concentration in H_2_O over time, 25 cm^3^ of ozonated water was collected at regular intervals and placed in 100 cm^3^ Erlenmeyer flasks into which 0.4 g of potassium iodide had previously been weighed. Then, 3 cm^3^ 0.1 M hydrochloric acid was added to each flask, along with 5% starch solution used as an indicator. The samples were then placed in a dark spot for 30 min and after that they were successively titrated with a standard solution of 0.002 M sodium thiosulphate until the dark blue colour disappeared. The calculation of the concentration of dissolved ozone was performed according to Józefczyk et al. [57].

#### 3.3.2. Spectrophotometric Method

To perform the measurement, the spectrum of the collected solution was recorded using a Biosens UV 5100 spectrophotometer (Biosens Marcin Guz, Warsaw, Poland). A portion of the 25 cm^3^ water solution sample was placed in a 1 cm thick cuvette and the spectrum between 190 and 350 nm was measured. The concentration of the O_3_ in the ozonated water was determined based on the molar absorption coefficient for the ozone wavelength of 260 nm, amounting to ε = 3300 L mol^−1^ cm^−1^ [58].

### 3.4. Measurement of Dissolved Anions in H_2_O

Water samples of 25 cm^3^ were subjected to analyses using ion chromatography (Dionex ICS-1000, Dionex; Titan Way Ste 1002 Sunnyvale, CA, USA), in accordance with the method described by Józefczyk et al. [57].

### 3.5. Antioxidant Potential, Polyphenol and Vitamin C Contents

The antioxidant potential of young barley plants was determined using an ABTS (2.2′-azino-bis-(3-ethylbenzothiazolin-6-sulfonic acid) test in accordance with the method presented by Matłok et al. [58]. The total polyphenol content in the raw material was measured using the Folin–Ciocalteu method, described by Matłok et al. [59]. The content of vitamin C in young barley leaves was determined using the method described by Piechowiak et al. [60].

### 3.6. Polyphenolic Compounds Profiles Analysis

Samples of plant material were prepared for chromatographic analyses according to the methodology described by Matłok et al. [61]. The determination of polyphenolic compounds was carried out using the ultra-performance liquid chromatography (UPLC) Waters ACQUITY system (Waters, Milford, MA, USA). The UPLC system was equipped with a binary pump manager, column manager, sample manager, photodiode array (PDA) detector and tandem quadrupole mass spectrometer (TQD) with an electrospray ionisation (ESI) source. The separation of polyphenols was performed using a 1.7 µm, 100 mm × 2.1 mm UPLC BEH RP C18 column (Waters, Milford, MA, USA). For the anthocyanin investigation, the mobile phase consisted of 2% formic acid in water, *v*/*v* (solvent A) and 2% formic acid in 40% acetonitrile, *v*/*v* (solvent B). However, in the case of other polyphenolic compounds, water (solvent A) and 40% acetonitrile, *v*/*v* (solvent B) were used. The flow rate was kept constant at 0.35 mL/min for a total run time of 8 min. The system was run with the following gradient program: from 0 min 5% B, from 0 to 8 min linear to 100% B and from 8 to 9.5 min for washing and back to initial conditions. The injection volume of the samples was 5 µL, and the column was supported at 50 °C. The following TQD parameters were used: cone voltage of 30 V, capillary voltage of 3500 V, source and desolvation temperature 120 °C and 350 °C, respectively, and desolvation gas flow rate of 800 L/h. Characterisation of the individual polyphenolic compounds was performed on the basis of the retention time, mass-to-charge ratio, fragment ions and comparison with data obtained with commercial standards and literature findings. Obtained data were processed in Waters MassLynx v.4.1 software (Waters, USA) [61]. The method was validated for parameters such as linearity, accuracy (relative error, RE), limit of detection (LOD), limit of quantification (LOQ) and precision (relative standard deviation, RSD). Quantification was determined by the injection of solutions of known concentrations ranging from 0.05 to 5 mg mL^−1^ (R^2^ ≤ 0.999) of the following phenolic compounds as standards: caffeic acid, p-coumaric acid, ferulic acid, luteolin 7-*O*-gluoside and apigenin 8-*C*-glucoside (vitexin) (Extrasynthese, Genay Cedex, France). Stock standard solutions of the five polyphenols were prepared with methanol. Six calibrators established the peak area ratio of each polyphenol versus the nominal concentration. The regression equation was obtained by a weighted (1/c2) least-squares linear regression. The LOD was determined as a signal-to-noise ratio (S/N) of 3:1, and the LOQ was determined as a S/N of >10. An acceptable RE within ±20% and the intra- and inter-day variations were determined using relative standard deviation (RSD) values, which were <3.5% for all the analysed compounds.

### 3.7. SOD, CAT, PPO and PAL Activity

The activity of SOD, CAT, GPOX, PPO and PAL enzymes in young barley leaves was measured according to the methods described by Matłok et al. [4].

### 3.8. ROS Level Analysis

The levels of reactive oxygen species (ROS) generated in young barley plants irrigated with different types of H_2_O were measured according to the methodology described by Piechowiak et al. [60].

### 3.9. Relative Chlorophyll Content in Leaves (SPAD)

Relative chlorophyll contents in young barley leaves were measured using SPAD 502 (Konica-Minolta Inc., Osaka, Japan), in line with the method described by Matłok et al. [7].

### 3.10. Statistical Analysis

One-way analysis of variance (ANOVA) was conducted at a significance level of α = 0.05 using STATISTICA 13.1 software (TIBCO Software Inc., Hillview Avenue, Palo Alto, CA, USA). The mean values calculated from the three independent replications were analysed statistically by comparing the results between the variants of the experiment.

## 4. Conclusions

It was shown that ozonated water can successfully be used in the cultivation of young barley. This process, however, is more complicated than the physical dissolution of ozone in water and involves a series of subsequent reactions. Depending on the degree of saturation with ozone and the accompanying consecutive reactions, water produced different modulating effects in the biosynthesis of bioactive compounds in young barley, mainly small molecule antioxidants and vitamin C. It was shown that after the process of water saturation with gaseous O_3_, the latter is converted to compounds with high oxidative potential and good stability; these, in turn, lead to the oxidation of oxidates generated from atmospheric nitrogen into nitrates which exhibit fertilising properties. Thirty minutes after the process of H_2_O saturation with gaseous O_3_ was completed, the tests showed the highest concentrations of nitrates and a relatively high oxidative potential of the solution originating from H_2_O_2_ with a low concentration of the dissolved O_3_. This solution exhibited the highest activity modulating the biosynthesis of polyphenols, small molecule antioxidants and vitamin C. The resulting differences were significant, and they were reflected by 15% higher total polyphenol content, 35% higher antioxidative potential and 57% greater content of vitamin C compared to the control specimens (plants treated with fresh H_2_O).

## Figures and Tables

**Table 1:** The characteristics of water used in irrigation of barley plants.

Type of Water	pH	Iodometric Concentration of O_3_ [ppm]	Spectrophotometric Concentration of O_3_ [ppm]	Oxidizability [mmol S_2_O_4_^−^]
V1	8.0	0	0	0
V2	8.4	7.68	3.0	0.008
V3	8.5	4.80	0.5	0.005

**Table 2:** Individual phenolic compounds identified by UPLC-PDA-MS/MS in barley leaves.

Compound		Rt	λ_max_	[M − H]^−^ *m*/*z*		Content (%)		
		min	nm	MS	MS/MS	V1	V2	V3
1	*p*-coumaric acid	2.54	309	163	119	0.13 ^a^	1.20 ^b^	0.16 ^a^
2	Caftaric acid	2.66	288sh. 327	311	179	0.23 ^a^	0.91 ^c^	0.32 ^b^
3	Feruloyl-caffeic acid	3.05	288sh. 322	367	193	0.88 ^b^	0.42 ^a^	0.83 ^b^
4	Unspecified caffeic derivative	3.13	288sh. 324	425	367	0.44 ^a^	0.41 ^a^	0.42 ^a^
5	Luteolin 6-*C*-arabinoside-8-*C*-glucoside	3.22	274. 341	579	447. 285	0.36 ^a^	0.64 ^b^	0.35 ^a^
6	Isoscoparin 7-*O*-glucoside	3.32	276. 333	623	461. 299	0.65 ^b^	0.26 ^a^	0.58 ^b^
7	Isoorientin 7-*O*-glucoside	3.48	269. 347	609	447. 285	0.27 ^a^	0.33 ^a^	0.32 ^a^
8	Isovitexin 7-*O*-glucoside	3.60	269. 331	593	431	86.63 ^a^	83.93 ^a^	86.71 ^a^
9	Isovitexin 7-*O*-(6′-*p*-coumaroyl)-glucoside	3.67	271. 324	739	593	2.10 ^a^	3.71 ^b^	2.46 ^a^
10	Isovitexin	3.84	270. 338	431	269	0.64 ^a^	0.62 ^a^	0.63 ^a^
11	Isoorientin 7-*O*-(6″-sinapoyl)-glucoside	3.99	271. 338	815	447	0.74 ^b^	0.63 ^a^	0.61 ^a^
12	Isoorientin 7-*O*-(6″-feruloyl)-glucoside	4.26	271. 338	785	447	0.70 ^a^	0.75 ^a^	0.72 ^a^
13	Isovitexin 7-*O*-(6″-sinapoyl)-glucoside	4.57	271. 334	799	431	1.66 ^a^	1.88 ^b^	1.39 ^a^
14	Isovitexin 7-*O*-(6″-sinapoyl)-glucoside-4′-*O*-glucoside	4.67	270. 338	961	799. 593	0.43 ^b^	0.39 ^a^	0.34 ^a^
15	Isovitexin 7-*O*-(6″-feruloyl)-glucoside	4.77	271. 331	769	431	3.56 ^a^	3.41 ^a^	3.64 ^a^
16	Apigenin 6-*C*-arabinoside-8-*C*-glucoside	4.89	269. 329	563	443	0.57 ^a^	0.51 ^a^	0.54 ^a^
Total polyphenolic content [mg 100 g^−1^ f.m.]						15.61 ^a^	14.82 ^a^	18.07 ^b^

RT—retention time; sh = shoulder peak; ±SD and *n* = 3; FD. Note: differences between the results are marked with small letters; significance level is defined as *p* < 0.05.

## Data Availability

Not applicable.

## Sample Availability

Samples of the compounds are available from the authors as extracts collected during investigation.
